# Immunoglobulin G4-Related Disease Mimicking Meningioma and Dural Metastasis: A Case Report

**DOI:** 10.7759/cureus.89227

**Published:** 2025-08-01

**Authors:** Sho Nitta, Yuki Sakaeyama, Shuhei Kubota, Kota Yamaguchi, Nobuo Sugo

**Affiliations:** 1 Department of Neurosurgery, Toho University, Chiba, JPN; 2 Department of Neurosurgery, Toho University, Tokyo, JPN

**Keywords:** cerebral angiography, dural metastasis, igg4-related disease (igg4-rd), magnetic resonance spectroscopy (mrs), meningioma

## Abstract

Herein, we report a case of immunoglobulin G4-related disease (IgG4-RD) that required differentiation from meningioma and dural metastasis based on neuroradiological imaging results, particularly focusing on magnetic resonance spectroscopy (MRS) and cerebral angiography findings. The patient was a 67-year-old woman who presented with left lower-limb weakness. She had been diagnosed with breast cancer 11 years earlier and was treated with surgical resection, followed by hormone therapy. Gadolinium-enhanced brain magnetic resonance imaging revealed a well-defined, homogeneously enhanced lesion accompanied by a dural tail sign. MRS revealed an increased choline peak with a high choline/N-acetylaspartate ratio and an elevated combined lipid/lactate peak. Based on these findings, a metastatic brain tumor or a malignant meningioma was initially suspected. Cerebral angiography demonstrated poor vascularity within the lesion. The tumor was resected. Part of the dura mater was significantly thickened due to involvement of the lesion, which also firmly adhered to the cortical surface. Histopathological examination with hematoxylin and eosin staining revealed a remarkable proliferation of collagen fibers and dense lymphocyte and plasma cell infiltration. Immunohistochemical staining showed an IgG4-/IgG-positive plasma cell ratio of 45%, with elevated levels of IgG4-positive plasma cells (40-50 per high-power field). A typical storiform pattern of fibrosis was not clearly observed. However, the presence of marked fibrosis and dense infiltration of IgG4-positive plasma cells led to the diagnosis of IgG4-RD. Even if MRS shows a tumor-like pattern, the identification of poor vascularity on cerebral angiography should prompt the consideration of IgG4-RD as a differential diagnosis, as in the current case.

## Introduction

Immunoglobulin G4-related disease (IgG4-RD) is a systemic condition characterized by immune-mediated inflammation and mass-forming lesions involving multiple organs, marked by infiltration of immunoglobulin G4 (IgG4)-positive plasma cells and fibrosis. Representative manifestations include autoimmune pancreatitis and retroperitoneal fibrosis, and early corticosteroid therapy can prevent progression to irreversible fibrotic changes [1,2]. Intracranially, it can involve the dura mater, pituitary gland, and orbit [1,2]. On neuroimaging, intracranial IgG4-RD often appears as a contrast-enhanced mass adjacent to the dura mater, closely resembling the radiological features of a meningioma [2]. Dural metastases, which may originate from malignancies such as breast, prostate, and renal cancer, can also present as localized masses on imaging, mimicking meningiomas [3]. Considering these similarities, neuroradiological differentiation among IgG4-RD, meningioma, and dural metastasis remains a diagnostic challenge [4,5].

Therefore, in the differential diagnosis using neuroradiological imaging, modalities such as magnetic resonance imaging (MRI), magnetic resonance spectroscopy (MRS), cerebral angiography, and single-photon emission computed tomography are often utilized, and the diagnosis is made based on a comprehensive assessment of the findings [6-10].

To date, only a limited number of reports have explicitly detailed MRS or cerebral angiography findings in IgG4-RD [11].

Gross total resection is desirable for meningiomas and metastatic brain tumors when feasible; however, in IgG4-RD, complete resection is not always required, as corticosteroid therapy is the mainstay of treatment. Therefore, making this differential diagnosis preoperatively is clinically important for determining subsequent therapeutic strategies.

Herein, we report a case of IgG4-RD in a patient with a history of breast cancer, presenting as a localized, dura-based mass on gadolinium-enhanced MRI, which required differentiation from meningioma and dural metastasis. In addition, we describe the findings of MRS and cerebral angiography, which are part of the diagnostic evaluation.

## Case presentation

**Figure 1:**
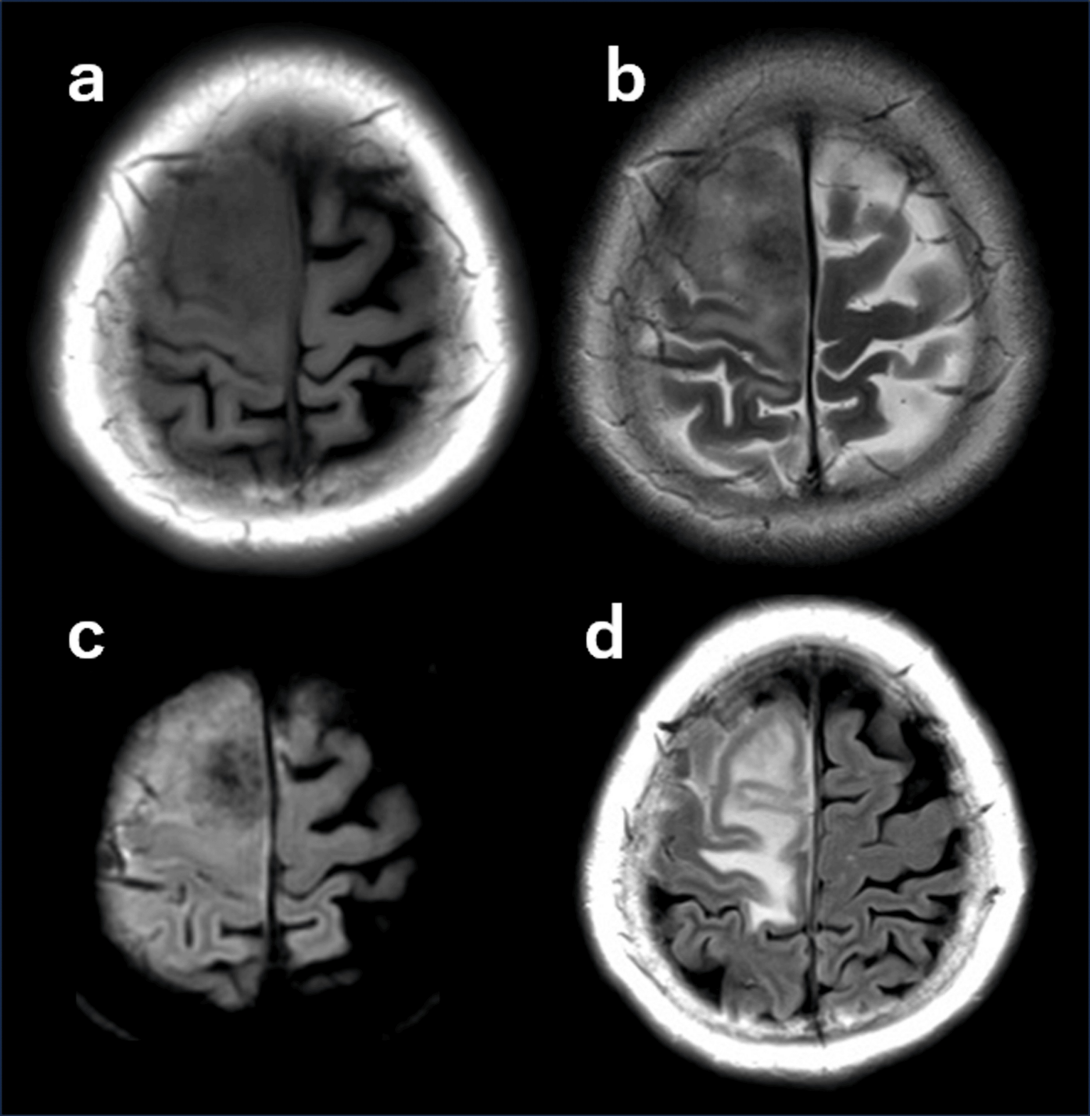
Preoperative magnetic resonance imaging. (a) T1-weighted image. (b) T2-weighted image. (c) Diffusion-weighted image. (d) Fluid-attenuated inversion recovery.

A 67-year-old female patient presented with left lower-limb weakness. Neurological examination revealed motor paresis in the left lower extremity, with a manual muscle test grade of 4. The patient’s medical history was remarkable due to the diagnosis of breast cancer 11 years earlier, which was treated with surgical resection, followed by hormone therapy. Brain MRI revealed a lesion in the right frontal region, which had an iso- to hypointense appearance on T1-weighted images and a hypointense appearance on T2-weighted images (Figures 1A, 1B). Diffusion-weighted imaging revealed areas of hypointensity within the lesion (Figure 1C). Meanwhile, fluid-attenuated inversion recovery images showed prominent perilesional edema (Figure 1D).

**Figure 2:**
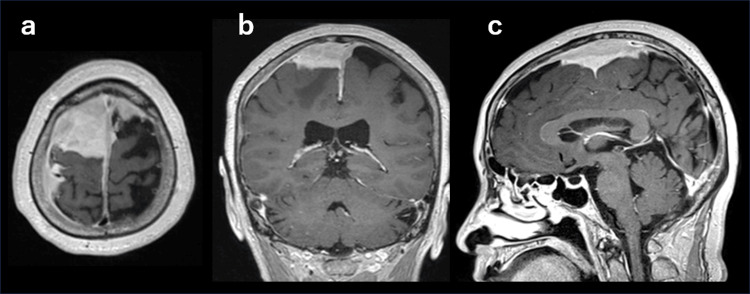
Gadolinium-enhanced magnetic resonance imaging. (a) Axial view. (b) Coronal view. (c) Sagittal view.

On gadolinium-enhanced T1-weighted images, the lesion appeared well-defined and homogeneously enhanced. Further, a dural tail sign was observed (Figure 2).

**Figure 3:**
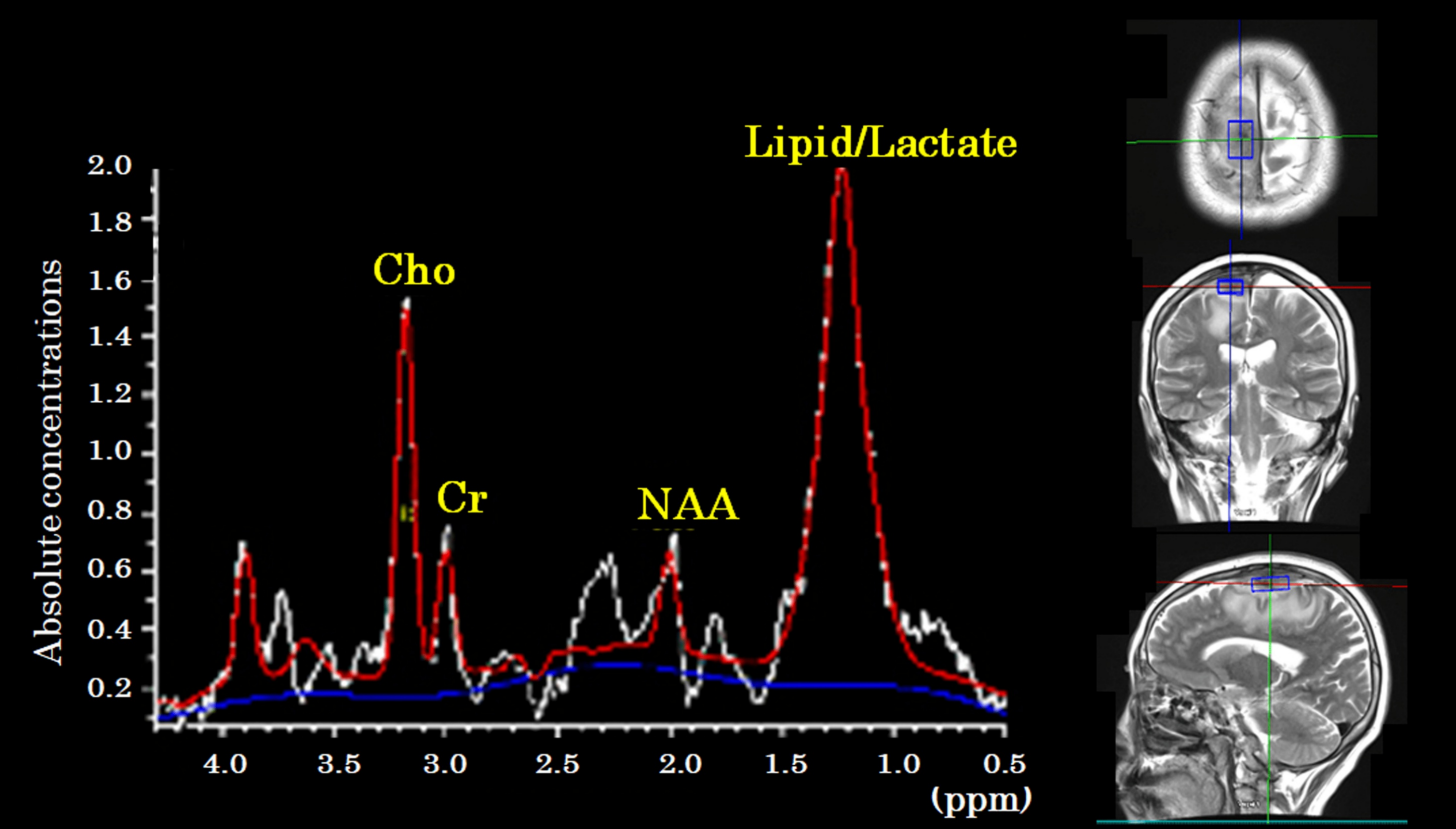
Magnetic resonance spectroscopy. Cho: choline; Cr: creatine; NAA: N-acetylaspartate.

Considering the patient’s history of breast cancer, dural metastasis was suspected, and preoperative MRS was performed. MRS revealed an elevated choline (Cho) peak, a high Cho/N-acetylaspartate (NAA) ratio, and an increased combined lipid/lactate peak (Figure 3).

**Figure 4:**
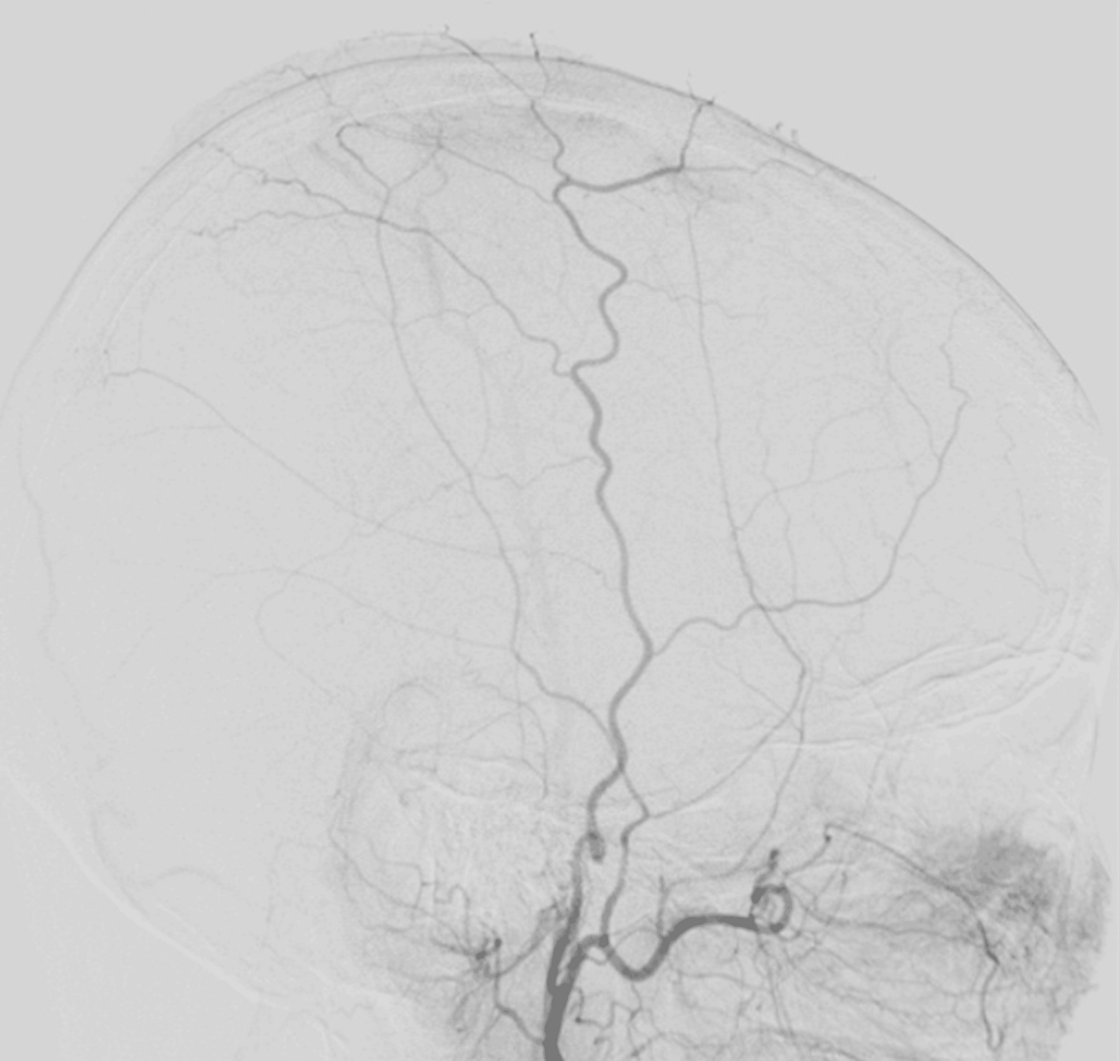
Right external carotid angiography.

Based on these findings, a metastatic brain tumor or a malignant meningioma was initially suspected. However, cerebral angiography demonstrated poor vascularity of the lesion (Figure 4). Serum IgG4 levels were not measured preoperatively.

Surgical resection of the tumor was performed. Intraoperatively, part of the dura mater was significantly thickened due to involvement of the lesion, which also firmly adhered to the cortical surface. Intraoperative frozen sections revealed fibrous connective tissue with lymphocytic infiltration, ruling out metastatic breast carcinoma and meningioma.

**Figure 5:**
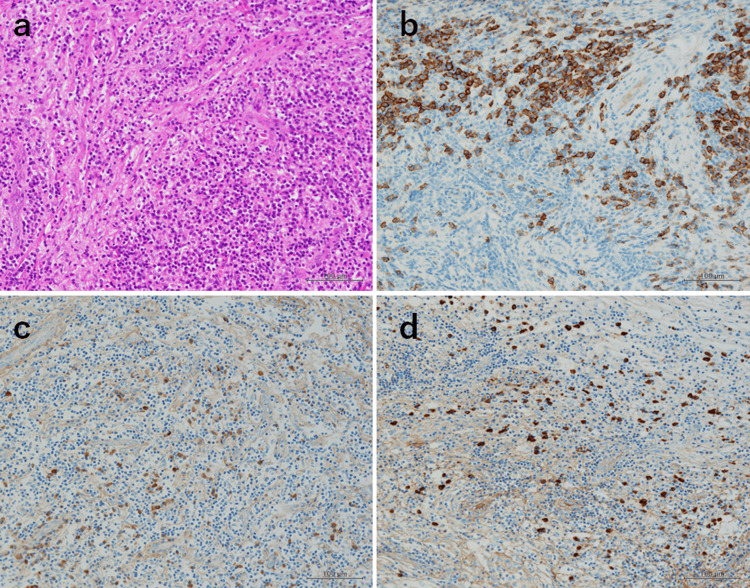
Histopathological examination. (a) Hematoxylin and eosin staining. (b–d) Immunohistochemical staining: (b) CD138, (c) IgG, and (d) IgG4.

Histopathological examination with hematoxylin and eosin staining revealed remarkable proliferation of collagen fibers and dense infiltration of lymphocytes and plasma cells (Figure 5A). Immunohistochemical analysis revealed a mixture of CD3-, CD5-, and CD20-positive lymphocytes, with no evidence indicative of a neoplastic process. Numerous CD138-positive plasma cells infiltrated the lesion (Figure 5B). The IgG4/IgG-positive plasma cell ratio was 45%. The number of IgG4-positive plasma cells was elevated at 40-50 per high-power field (Figures 5C, 5D). The typical storiform pattern of fibrosis, obliterative phlebitis, and eosinophils was not observed. However, based on the imaging findings, marked fibrosis, and dense infiltration of IgG4-positive plasma cells, a diagnosis of IgG4-RD was made.

Postoperative MRI demonstrated that the lesion had been almost completely resected, with only a small residual component remaining; therefore, a short course of corticosteroid therapy (10 mg/day orally) for three weeks was administered.

The patient’s left lower limb motor weakness improved, and he was discharged in an ambulatory condition. A whole-body computed tomography scan revealed no additional lesions suggestive of IgG4-RD. Follow-up was planned using contrast-enhanced MRI.

## Discussion

IgG4-RD is diagnosed based on three histopathological hallmarks: infiltration of IgG4-positive plasma cells, storiform fibrosis, and obliterative phlebitis [1]. In particular, infiltration of IgG4-positive plasma cells is essential in obtaining a diagnosis, with diagnostic criteria typically requiring >10 IgG4-positive cells per high-power field and an IgG4/IgG plasma cell ratio >40% [5]. Fibrosis is often observed in dural brain lesions and is occasionally accompanied by mild eosinophilic infiltration [1]. However, not all three histopathological features are necessarily present, and cautious evaluation of the fibrosis pattern and inflammatory cell localization can help differentiate IgG4-RD from meningioma [4,12]. In this case, marked fibrosis and IgG4-positive plasma cell infiltration were observed, leading to the diagnosis of IgG4-RD.

In Japan, the estimated prevalence of IgG4-RD is approximately six cases per 100,000 population [4]. Among these, involvement of the central nervous system accounts for less than 2% of all cases, making it an exceptionally rare manifestation [5]. On neuroradiological imaging, IgG4-RD often presents with MRI findings that closely resemble those of meningioma [2]. In the current case, the lesion exhibited homogeneous enhancement with a dural tail sign, making it radiologically indistinguishable from meningioma. Moreover, if lesions are located at dural junctions, particularly in regions commonly affected by meningiomas, such as the skull base and falx cerebri, the risk of misdiagnosis is higher [1,4,5]. In terms of neurological presentation, IgG4-RD shows a slowly progressive course, which is also a characteristic of meningioma [13]. In this case, gadolinium-enhanced MRI revealed a localized mass in the right frontal region. Considering the patient’s history of breast cancer, dural metastasis was suspected, and preoperative MRS was performed.

A critical evaluation of the specificity of MRS findings for IgG4-RD compared to neoplasms highlights several important limitations. MRS is a useful tool for differentiating neoplastic from non-neoplastic lesions [6,7]. Neoplastic lesions are typically characterized by a marked elevation of the Cho/NAA and Cho/creatine ratios, accompanied by lactate and lipid peaks [6,7]. In contrast, inflammatory lesions generally demonstrate only mild Cho elevation with preservation of NAA levels [7]. In meningiomas, an elevated Cho peak and the presence of an alanine peak are frequently observed, although the latter may be obscured by overlapping lactate signals, requiring cautious interpretation [14]. Reports of MRS findings in IgG4-RD are scarce. In one case, a significant decrease in the NAA/Cr ratio was noted, but the underlying mechanism was not clearly elucidated [11].

In the current case, MRS showed an elevated Cho/NAA ratio as well as lactate and lipid peaks. These findings made it difficult to distinguish the lesion from a neoplasm. An elevated Cho peak in IgG4-RD may reflect increased cell membrane synthesis due to the proliferative activity of plasma cells, lymphocytes, and fibroblasts. It is also suggested that proinflammatory cytokines under chronic inflammation enhance cell membrane metabolism. IgG4-RD remained on the list of differential diagnoses despite MRS demonstrating features typically seen in neoplastic lesions.

Meningiomas and dural metastases often present with a rich vascular supply on cerebral angiography [8,9]. Numerous intratumoral vessels are often observed in meningiomas, frequently leading to significant intraoperative bleeding. Therefore, preoperative embolization is commonly performed [8]. In contrast, cerebral angiography in this case revealed poor vascularity within the lesion, differing from the typical findings observed in meningiomas and dural metastases. The poor vascularity of IgG4-RD on cerebral angiography is assumed to be caused by its inflammatory and fibrotic nature, rather than a neoplastic process, leading to minimal neovascularization and limited vascular proliferation [1]. To our knowledge, this is one of the few instances showcasing cerebral angiography findings in intracranial IgG4-RD. Such results may be useful for differentiating IgG4-RD from meningioma and dural metastasis. If such MRS and cerebral angiography findings are observed, IgG4-RD should be considered. A comprehensive evaluation, including serum IgG4 measurement and steroid responsiveness assessment, is essential [15].

As limitations in the diagnosis of this case, the lack of serum IgG4 testing and the absence of long-term follow-up should be noted.

## Conclusions

Based on these findings, IgG4-RD should be considered even when MRS findings demonstrate features typically seen in neoplastic lesions.

This case highlights the diagnostic challenge of IgG4-RD when it presents as a solitary dural-based mass mimicking meningioma or dural metastasis. While MRI and MRS suggested a neoplastic lesion, cerebral angiography revealed poor vascularity, which raised suspicion for a non-neoplastic process. Histopathological findings, including dense IgG4-positive plasma cell infiltration and fibrosis, confirmed the diagnosis. The discrepancy between tumor-like imaging features and poor vascularity may serve as a useful clue for differentiating IgG4-RD. A multimodal diagnostic approach is essential for accurate diagnosis and appropriate management.
